# Diet Cost and Affordability in Queensland: A Two-Year Cross-Sectional Study

**DOI:** 10.3390/ijerph23040535

**Published:** 2026-04-20

**Authors:** Renae Earle, Tessa Kenney, Kora Uhlmann, Meron Lewis, Nicola Malone, Martin O’Flaherty, Simone Nalatu

**Affiliations:** 1 Health and Wellbeing Queensland, Brisbane, QLD 4064, Australia; uqrearle@uq.edu.au (R.E.); tessa.kenney@hw.qld.gov.au (T.K.); kora.uhlmann@hpw.qld.gov.au (K.U.); nicola.malone@hw.qld.gov.au (N.M.); 2 Faculty of Health Sciences and Medicine, Bond University, Robina, QLD 4226, Australia; 3 Faculty of Health, Medicine and Behavioural Sciences, The University of Queensland, St. Lucia, QLD 4072, Australia; m.lewis@uq.edu.au (M.L.); m.oflaherty@uq.edu.au (M.O.)

**Keywords:** food affordability, food cost, rural and remote, Queensland, diet affordability, food insecurity, monitoring and surveillance

## Abstract

**Highlights:**

**Public health relevance—How does this work relate to a public health issue?**

- Diet cost and affordability are important, yet under-monitored determinants of food security in Queensland.
- Food security is an International Human Right and critical to good health and wellbeing.
- Remote communities face compounding food security challenges (including geographic isolation, high freight costs and limited retail competition) which intensify diet-related cost-of living pressures and health inequity.

**Public health significance—Why is this work of significance to public health?**

- This study monitored diet cost and affordability across six urban, regional and remote Queensland regions over two years (2023 and 2024) using a standardized and evidence-based protocol.
- In 2024, healthy diets in remote Far North Queensland became significantly cheaper, which could be associated with a cost-of-living policy intervention.

**Public health implications—What are the key implications or messages for practitioners, policy makers and/or researchers in public health?**

- Fiscal support can be effective in equalizing diet costs between remote and non-remote Queensland regions.
- Inequities related to diet affordability among low-income earners persist and highlight the need for sustained, coordinated and multi-strategic food security action.

**Abstract:**

Diet affordability is a critical determinant of food security, health and wellbeing. However, the cost and affordability of diets have not been routinely measured in Queensland (Australia) in over a decade. This study assessed the cost and affordability of healthy (based on national healthy eating guidelines) and habitual (less healthy, based on national reported intake) diets across six Queensland regions. Data were collected in 35 communities, over two years (2023 and 2024), using the evidence-based Healthy Diets Australian Standardised Affordability and Pricing protocol. Data were analyzed relative to a six-person intergenerational Aboriginal and Torres Strait Islander reference household. Results indicate that, across Queensland, healthy diet costs are above the threshold for food stress in Aboriginal and Torres Strait Islander households. On average, healthy diets were 30% cheaper than the habitual diet (which include alcohol and takeaway foods) but cost at least 26% of household income (above the 25% threshold for food stress). In 2023, healthy diets were on average 31% more expensive in remote communities compared to urban and regional centers. In 2024, the cost of a healthy diet in remote communities decreased significantly by 24%, narrowing diet cost differences between remote and non-remote regions. This shift could be associated with the implementation of a freight subsidy in remote Queensland, or other influences on remote food pricing. Findings highlight diet-related cost-of-living challenges for Aboriginal and Torres Strait Islander families, underscore the need for ongoing monitoring and provide insight for policy interventions (such as targeted subsidies) to improve diet affordability and reduce nutrition-related health inequity.

## 1. Introduction

Under the Universal Declaration of Human Rights, food security is a human right. It occurs when “all people, at all times, have physical, social, and economic access to sufficient, safe, and nutritious food that meets cultural preferences and dietary requirements for a healthy life” [1]. The drivers of food insecurity are complex and varied; the affordability of food is one critical determinant [2,3]. High food costs, paired with low income, can compromise food security and negatively impact physical health, mental wellbeing, and social outcomes [4,5]. Poor nutrition, related to food insecurity, is associated with an increased risk of chronic disease, impaired child development, limited daily functioning, and weakened social connection [3,6,7,8,9,10].

These issues are amplified by rising cost-of-living pressures, which (among many other factors) can disproportionately impact those living in remote areas and Aboriginal and Torres Strait Islander peoples. Prior to colonization, Aboriginal and Torres Strait Islander food practices protected the health of communities, culture and the natural environment. The forcible disruption of traditional food systems has resulted in reliance on Western food systems, which are struggling to meet the needs of remote communities [11].

Approximately 41% of Australian Aboriginal and Torres Strait Islander households experience food insecurity [12]. Aboriginal and Torres Strait Islander peoples, larger (group) households, socioeconomically disadvantaged communities and those living in remote areas are more likely to experience food insecurity compared to the rest of the Australian population [12,13,14,15]. Diet affordability plays a critical role in these disparities and is determined by diet cost and household income [16]. Food items may be objectively inexpensive yet remain unaffordable for households with limited financial resources [17]. Healthy diets are often unaffordable for people on low income [18,19]. Further, factors associated with remote living (geographic isolation, high freight and transport costs, limited food retail options, and reduced employment opportunities) intensify food security challenges [20]. These inequities in diet affordability (among others) actively shape local food environments, nutrition behaviors, diet quality, and ultimately, health and wellbeing [4,10,18,21,22,23,24,25]. Despite this, research on diet affordability and food security in Queensland, Australia, is limited [17].

Previous food affordability monitoring has provided valuable insights about food security pressures [26]. However, in Queensland, state-wide efforts to monitor diet cost are over a decade old [26]. Furthermore, regional and remote communities have often been underrepresented in previous monitoring, despite being at increased risk of food insecurity [26]. This study, therefore, aimed to monitor diet cost and affordability across urban, regional, and remote regions of Queensland between 2023 and 2024.

## 2. Materials and Methods

### 2.1. Design and Setting

Using an evidence-based protocol, the Healthy Diets Australian Standardised Affordability and Pricing (Healthy Diets ASAP), primary data were collected over two years across six Queensland regions, with a focus on Far North Queensland (FNQ) [27]. The use of this consistent, evidence-based methodology allows for meaningful comparisons across time and geography, thus providing valuable insights for monitoring diet affordability and informing public health policy [27,28]. This cross-sectional study was conducted over two consecutive years (2023 and 2024).

FNQ was a focus for this study due to its remoteness and increased food security and diet cost challenges, which is recognized by government policy and extensive previous research [29,30]. Diet costs were monitored across four FNQ remote regions—Cape York (CY), Lower Gulf (LG), Inner Torres Strait Islands and Northern Peninsula Areas (TSI/NPA) and Outer Torres Strait Islands (OTSI)—and two non-remote regions: Cairns (regional) and Brisbane (urban). Remote regions were defined based on known supply chain patterns derived from previous mapping [31].

### 2.2. Healthy Diets ASAP

The Healthy Diets ASAP protocol, modified for Aboriginal and Torres Strait Islander groups, was utilized in all data collection and analysis processes [28]. The protocol, developed by Lee and colleagues, enabled consistent, place-based assessments of diet cost and affordability relative to two diet types: healthy and habitual [27,28]. As per the modified Healthy Diets ASAP protocol, the healthy diet was based on the 2013 Australian Dietary Guidelines, and the habitual diet (less healthy) was based on dietary intake data from the Australian Aboriginal and Torres Strait Islander Health Survey (2012–2013) [27,28,32,33]. The protocol outlines an agreed-upon list of standardized food/drink items that were reported as commonly consumed in national dietary intake surveys, and are often available in remote and urban stores, to guide data collection about food/drink prices [28]. This methodology has been used previously in FNQ Aboriginal and Torres Strait Islander communities and was selected for its suitability to this study context [28,34].

For the purposes of this study, diet cost calculations were performed relative to Healthy Diets ASAP Household 1—a six-person intergenerational Aboriginal and Torres Strait Islander household consisting of one senior female (71+ years), one adult male and one adult female (31–50 years), and three children (aged 14, 8, and 4 years). This household was selected as previous research has demonstrated that Aboriginal and Torres Strait Islander households, as well as larger (group) households, are at increased risk of food insecurity [12,14,15,35]. Further, in discussion with local stakeholders and based on national surveillance data, an Aboriginal and Torres Strait Islander household structure was considered to be the most representative of the remote FNQ communities included in this study [36].

### 2.3. Data Sampling

This study employed purposive and convenience sampling techniques. In non-remote locations (Brisbane and Cairns), two communities (one high- and one low-socioeconomic community) were purposively sampled. In remote regions, communities were sampled using convenience methods. The remote communities sampled in this research reflect areas where the authors had local partnerships that could be utilized for data collection and where there were available/existing staff to collect data. No communities (within the six regions of focus) were excluded from the study. Non-participating communities include those for which there were no possible mechanisms for data collection within the study timeframe (for example, no local workforce identified to undertake data collection, no local partnerships to support data collection, and/or no planned travel by authors and/or their colleagues to the community that could be utilized to collect data). Where possible, the same communities and stores were sampled between years.

As per the Healthy Diet ASAP protocol, data were collected in communities from local supermarkets, liquor stores and takeaway outlets (such as burger shops, fish-and-chip shops, pizzerias, and bakeries) [27,28]. Where there was more than one supermarket in a community (for example, in urban and regional locations), a cross section of supermarket types was sampled. For example, if two major supermarket chains were present in the community, both were sampled. If the community was dry or did not have any takeaway outlets (for example, some remote communities), no data related to alcoholic drinks or takeaway items were recorded for these communities. In these cases (for missing or unavailable food/drink items at the community level), the regional average price for these items was substituted.

### 2.4. Data Collection and Management

Food and drink price data were collected within communities from local food/drink outlets between September and December of 2023 and 2024. Data collection was conducted in accordance with the Healthy Diets ASAP protocol [27,28] utilizing three methods: in-person, online, and through store groups’ central data systems.

Using the online shopping platforms of major food/drink retailers, location-specific food and drink price data were collected. Through partnering with two remote retail groups, the research team also collected location-specific food/drink price data from the store group’s centralized food/drink pricing systems. Online and centralized data collection methods reflect the actual price of food/drinks to consumers at the point of sale and are therefore most accurate. Hence, wherever possible, these data collection methods were prioritized. In-person data were collected by personally visiting the food/drink outlet and manually recording the food and drink prices reflected on the shelf ticket. Permission from the local store manager was sought prior to collecting in-person data. Initially, the research team was concerned about the potential for in-person data collection to introduce error because shelf-ticket prices (particularly in remote stores with workforce shortages) are not always up to date (particularly for items that fluctuate in price frequently such as produce). However, preliminary testing of in-person and centralized data collection techniques in remote stores found that most discrepancies arising between these methods were small and non-significant. Hence, the possibility of systematic bias was ruled out. The data were primarily collected by the authors (RE, KU, NM, SN), other Health and Wellbeing Queensland colleagues, and local partners (for example, officers in local governments or community-controlled health organizations). University placement students (from public health and related disciplines) also supported data collection, particularly in urban and regional areas. All data collectors underwent training about the Healthy Diets ASAP protocol and were supported by ML, RE, and KU in data collection processes.

Data were entered and validated within the Healthy Diets ASAP online portal. As per the Healthy Diets ASAP protocol, if the price of a food/drink item could not be collected in a region (due to it being unavailable/missing in that region), price data from the nearest regional center were substituted [27,28]. Outliers were discussed among the research team and (where required) with data collectors before being resolved. A random sample of data (approximately 20%) underwent cross-checking for quality assurance purposes by one store group and the research team (RE, NM, TK). Discrepancies were discussed with store groups (where necessary) and resolved in collaboration with the research team.

### 2.5. Data Analysis

Using food and drink price data, diet costs were automatically analyzed and exported to Microsoft Excel (Microsoft, Redmond, DC, USA, version 16.107.4) from the Healthy Diets ASAP Portal [27,28,37]. The tool calculated healthy and habitual (less healthy) diet costs for a six-person Aboriginal and Torres Strait Islander Household per community. Communities were grouped based on the six pre-defined regions (CY, LG, OTSI, TSI/NPA, Brisbane, Cairns) to generate regional averages ($AUD per fortnight). Individual community-level results will not be publicly shared by this study in the interest of protecting the anonymity of participating communities and stores. All reporting and analysis were completed at the regional level.

To assess affordability, two levels of location-specific household income data were obtained in accordance with the Healthy Diets ASAP protocol [27]. Household income based solely on applicable welfare payments was estimated using Department of Human Services data [38]. For these payments, it was assumed that adults in the household were unemployed, and the senior adult received the age pension. Median household incomes were derived from the 2021 Australian Bureau of Statistics Census data for each local government area (community-level data) using the Census All Persons QuickStats function [39]. A full list of assumptions is included in Supplementary Table S1. Community-level income data were grouped based on the six pre-defined regions (CY, LG, OTSI, TSI/NPA, Brisbane, Cairns) to generate a regional average ($ income per household per fortnight). The regional average was then adjusted for wage growth in alignment with the Wage Price Index [40,41]. Household incomes were input into the Healthy Diets ASAP tool to calculate diet affordability metrics at a regional level (diet cost as a % of household income). Diet affordability metrics were compared against two thresholds: food stress and food affordability (representing diet cost >25% and >30% of household income, respectively) [5,42,43]. These thresholds are internationally recognized and have been consistently utilized in Australian literature about diet affordability [5,42,43]. These thresholds represent the point at which diet cost and household income are out of balance, potentially resulting in pressure on household finances to meet essential needs (for example, housing, medical, education, utilities) while maintaining the diet.

Preliminary testing revealed that diet cost variation within remote (CY, TSI/NPA, OTSI, LG) and non-remote (Cairns, Brisbane) regions was not statistically significant. Regions were therefore grouped by remoteness (remote or non-remote) for further statistical analysis. Linear regression with a three-way interaction between years (2023, 2024), diet types (healthy, habitual), and regions (remote, non-remote) was used to study differences or changes in cost. Cluster-robust standard errors were used to account for clustering at the community level [44]. Post hoc tests were used to quantify and test specific contrasts. Estimation was performed in Stata 19 (StataCorp LLC, College Station, TX, USA) using the regress and margins commands [45].

### 2.6. Ethics

This study was exempted from human research ethics review as it does not involve human participant data, and all data were publicly available.

### 2.7. Positionality Statement

This research was conducted in response to cross-sector consultation about food security in remote Queensland [29]. Through consultation with communities, diet cost and affordability were strongly raised by stakeholders (including Aboriginal and Torres Strait Islander peoples) as issues of importance. The research question explored by this study was therefore directly influenced by the voice of the community and other regional stakeholders.

It is acknowledged that the identity, beliefs, and values of authors influence many elements of research, including the questions asked and the interpretation and dissemination of findings. The research team is a combination of Torres Strait Islander (NM), non-Indigenous Australian (RE, KU, ML, MO, SN) and United States of America (TK) authors. The lead author (RE) is a non-Indigenous woman who has worked with remote Aboriginal and Torres Strait Islander communities and colleagues for six years. She has spent time in various FNQ communities, including those in which data were collected for this study. The wider authorship team occupies various positions about this research, including authors with lived experience and significant expertise working within remote Aboriginal and Torres Strait Islander communities.

Throughout the project, care was taken to engage in reflective practice, seek feedback from Aboriginal and Torres Strait Islander colleagues, and incorporate strengths-based and decolonizing language. To achieve this, research methods and dissemination products have been shared with Aboriginal and Torres Strait Islander colleagues and other organizations that represent key stakeholders for advice. Appropriate community dissemination products have been developed and shared with relevant community stakeholders.

## 3. Results

### 3.1. Sample Characteristics

Over two years, data were collected in 85 food/drink outlets across 35 communities. See Table 1. Compared to Brisbane and Cairns, a lesser variety of food/drink retail outlets were sampled in remote regions due to their general absence in small communities. Further details about the sample make-up can be found in Supplementary Table S2. Most of the data (61%) was collected in person, one third (30%) was provided electronically through stores’ centralized data systems, and a small proportion of data (9%) was collected online.

### 3.2. Diet Cost

**Figure 1:**
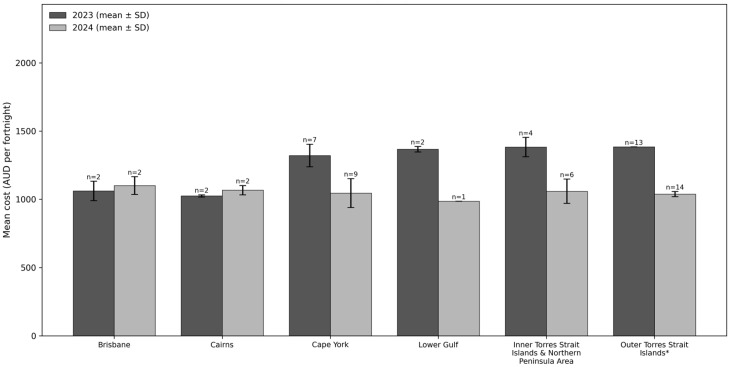
Mean fortnightly cost of a healthy diet in Queensland for a six-person Aboriginal and Torres Strait Islander household (*n* = 35). * In 2023, *n* = 13 samples were collected centrally from one store group operating in the region. For this store group, all stores in the region have the same pricing structure (i.e., prices are guaranteed across stores). Therefore, the standard deviation is zero.

**Figure 2:**
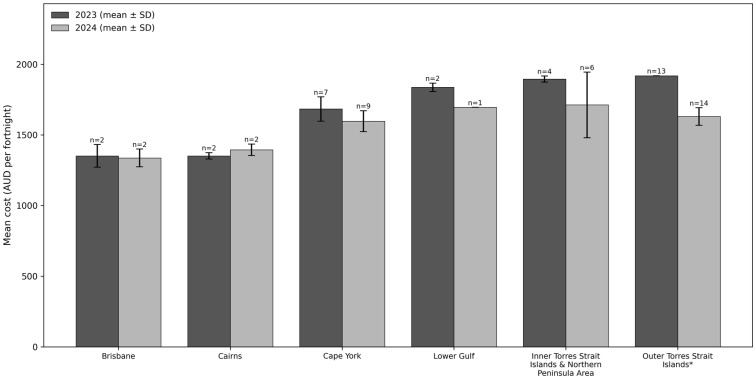
Mean fortnightly cost of a habitual (less healthy, including alcohol and takeaway food) diet in Queensland for a six-person Aboriginal and Torres Strait Islander household (*n* = 35). * In 2023, *n* = 13 samples were collected centrally from one store group operating in the region. For this store group, all stores in the region have the same pricing structure (i.e., prices are guaranteed across stores). Therefore, the standard deviation is zero.

The mean costs of healthy and habitual diets are reported in Figure 1 and Figure 2, respectively. In both years and across all regions, the habitual diet (including alcohol and takeaway food) was 30% more expensive than the healthy diet (average habitual diet cost = $1686.10 (AUD) per fortnight vs. average healthy diet cost = $1176.30 per fortnight).

In 2023, both diets were significantly more expensive in remote (CY, LG, OTSI, TSI/NPA) compared to non-remote (Cairns and Brisbane) regions. In 2023, the healthy diet was, on average, 31% ($323.80 per fortnight) more expensive in remote compared to non-remote regions.

In 2024, the Queensland Government’s Remote Communities Freight Assistance Scheme (RCFAS) was introduced in remote regions only [46]. In participating stores (remote supermarkets), the RCFAS applied a 20% discount on all food and drink prices, except for alcohol and sugar-sweetened soft drinks. Healthy diet cost in remote regions was 24% ($321.80 per fortnight) less in 2024 compared to 2023. This diet cost decrease was not observed in non-remote regions, where the cost of a healthy diet remained relatively stable ($38.70 per fortnight more expensive in 2024 compared to 2023) (the RCFAS was not applied in non-remote regions). In 2024, healthy diets were approximately the same cost across remote and non-remote regions ($36.70 per fortnight less in remote compared to non-remote regions on average).

In 2024, the habitual diet also reduced in cost in remote regions (compared to 2023), but to a lesser extent than healthy diets. In 2024, the habitual diet cost in remote regions was 11% ($209.70 per fortnight) less compared to 2023. Similar to the trends in healthy diet cost, the cost of the habitual diet remained relatively stable in non-remote regions ($26.70 per fortnight more expensive in 2024 compared to 2023). As a result, in 2024, the habitual diet remained significantly more expensive in remote regions compared to non-remote regions ($268.90 more per fortnight).

Table 2 shows differences in diet cost across diet type, region, and year derived from the linear regression analysis. Results indicate that the cost difference between healthy and habitual diets was substantially larger in remote areas than in non-remote areas, and this cost difference increased in remote areas between 2023 and 2024.

### 3.3. Diet Affordability

The affordability of healthy and habitual diets is reported in Table 3. Results indicate that, across Queensland, for a six-person Aboriginal and Torres Strait Islander household, a healthy diet costs >25% of household income, exceeding the threshold for food stress. In many cases, diets cost >30% of household income, meaning they exceeded the threshold for affordability.

Diet affordability is a function of both household income and diet cost. Household income (median and welfare) estimates varied across regions, with median income being higher than welfare income in non-remote regions, while the inverse was true for remote regions. Accordingly, in remote regions, diets were more affordable on welfare income compared to the median income for six-person Aboriginal and Torres Strait Islander households. This was not the case for non-remote regions, in which diets were more affordable (or the same) on the median income (compared to welfare income). Further details about regional estimated median and welfare-only household income are reported in Supplementary Table S1.

## 4. Discussion

This study highlights the cost and affordability of healthy and habitual diets across Queensland in 2023 and 2024, with a particular focus on Aboriginal and Torres Strait Islander households. Across regions, healthy diets were less expensive than the habitual diet (inclusive of alcohol and takeaway food), which is consistent with the findings of other research utilizing the Healthy Diets ASAP protocol [3,47]. Despite being less expensive, the affordability of healthy diets exceeded the threshold for food stress. In 2024, the cost of healthy diets reduced significantly in remote Queensland compared to 2023. As a result, healthy diet cost differentials between remote and non-remote regions equalized. This is important because, over decades, research has demonstrated persistent inequities in the cost of food for remote communities [26,48].

Diet cost improvements in remote regions could (at least partially) be associated with the implementation of the Queensland Government’s RCFAS [46]. However, this study is cross-sectional and, therefore, is insufficient to identify causal factors explaining the 24% healthy diet cost decrease in remote regions between 2023 and 2024. As such, changes in diet cost cannot be solely attributed to the impact of the RCFAS, nor is this study an evaluation of the RCFAS—it does not capture other various challenges, opportunities, and impacts of the RCFAS. There are several other plausible explanations that could also have contributed to the observed changes, including, but not limited to, changes in remote retailer pricing strategies, global supply chain changes, demand on food supply or climate/weather events.

Results suggest that, without support, household diet costs for a six-person household are more expensive in remote communities compared to non-remote regions [24]. Disparities in diet costs across Queensland are the result of complex and long-standing systemic food system barriers, including, but not limited to, high freight costs, limited market competition, inadequate transport infrastructure and frequent disruptive weather events [29,49,50]. Despite these challenges, remote retailers continue to provide essential grocery services where they are needed. Previous research has demonstrated that geographic remoteness and the type of food retail outlets are associated with diet cost [3,24]. In this study, non-remote regions (Brisbane and Cairns) were more likely to have multiple supermarkets and a variety of takeaway outlets (e.g., bakery, fish-and-chip shop, burger shop and liquor store). Conversely, in remote regions, there was usually one supermarket per community, a lesser variety of food/drink takeaway outlets, and, in some communities, no liquor store (due to community alcohol management plans) (see Supplementary Table S2). These factors may result in a less competitive market in remote communities (due to fewer food/drink retail outlets), contributing to higher diet costs in these regions (in 2023), among a variety of other factors. Further, in non-remote regions such as Brisbane and Cairns, data were collected from major national and international food retail chains (for example, McDonald’s, Woolworths, and Coles). Typically, these large retail chains have greater access to resources (such as scale, power and strategy) that enable them to price products competitively. Smaller remote community stores and independently operated retailers do not have access to the same level of resources, which can influence diet cost. This study suggests that the support of a subsidy (the RCFAS) to overcome the high costs of freight and limited market competition in remote communities could be associated with significant gains in diet costs in remote regions. Findings align with national and international evidence suggesting that a 20% discount can improve economic access to food [51,52]. Comparatively, in the absence of food pricing intervention (in non-remote regions), diet costs changed minimally. Slight diet cost increases observed in Brisbane and Cairns (an average of 3%) were approximately in line with national inflation rates [53].

Consistent with previous research utilizing the Healthy Diets ASAP tool, this study found the healthy diet to cost less than the habitual diet (on average $509.80 per fortnight less) [3,18,47]. However, this result must be interpreted with caution. Compared to the healthy diet, the habitual diet includes a greater number of food/drinks and expensive items such as takeaway food and alcoholic beverages (that are not included in the RCFAS) [27,28]. These items (takeaway foods and alcoholic beverages) make large contributions to the higher cost of the habitual diet. Across Australia, dietary patterns remain misaligned with the healthy diet [15]. Less than 5% (4.2%) of Australians consume the recommended daily serves of fruit and vegetables [54] Hence, the healthy diet investigated in this study is aspirational and highly unlikely in practice. Factors influencing diet choice are multi-faceted and not solely driven by finance [3]. Food availability, preferences, cooking facilities, perceived affordability, convenience, shelf stability, dietary needs, and cultural and social norms are important additional determinants of food choice (that are outside the scope of this study) [3]. For example, recent data suggests that 60% of Aboriginal and Torres Strait Islander peoples living in remote communities were not always able to access healthy and nutritious foods [15], meaning the healthy diet investigated in this study may be unavailable to remote households (regardless of its cost/affordability) [34,55]. This study does not capture the complementary resources required to achieve a healthy diet, which are also often less available in remote communities [23,31,34,56]. For example, the habitual diet contains convenience, ready-made items (such as takeaway foods and instant noodles), which require little to no resources for preparation and consumption. Healthy foods, on the other hand, often require additional household infrastructure and equipment (for example, water security, reliable electricity, white goods, working cooktops and appliances). Evidence suggests that large households in remote areas (such as those in focus for this study) are at increased risk of malfunctioning health hardware in their homes [57,58], which can be a barrier to good nutrition. Furthermore, consuming a healthy diet requires high levels of food literacy, including the resources, knowledge, skills and time to choose, prepare and budget for a healthy diet. In some communities (particularly remote communities with insufficient access to health services and food literacy programs), healthy diets may be out of reach (despite being less expensive than the habitual diet) [59]. Overall, these factors suggest that food price interventions, in isolation, are insufficient to support a healthy diet for all households and support existing literature that highlights diet cost as one of many influences on diet choice [3]. The promotion of healthy diets must be multi-strategic and target various determinants of diet choice, including housing/water security and food availability and quality, among others [60].

Results demonstrate that income remains an important factor in diet affordability. In non-remote communities, diets appear more affordable on median income compared to welfare. The inverse appeared to be true for non-remote regions. This is a result of different welfare and median income structures across remote and non-remote Queensland regions (refer to Supplementary Table S1). However, several limitations mean this finding should be interpreted with caution. Diet affordability findings with respect to median income are limited because median income estimations are taken from standard Census Data (gross income prior to tax payable) and are not specific to the Aboriginal and Torres Strait Islander reference household in focus for this study [40,41]. Due to inequitable systems and structures that reduce economic and employment opportunities for Aboriginal and Torres Strait Islander peoples, the reference household in focus for this study is more likely to be low-income [61]. Hence, utilizing standard Census data risks over-inflating the median household income for the reference Aboriginal and Torres Strait Islander household, therefore potentially failing to fully capture the extent of food affordability issues in this population (with respect to median income). Furthermore, the percentage of total households earning the median income may be low—the median income is a middle point of all household income in the region and does not necessarily reflect the income of most households. Median income calculated for this study is not reflective of the income distributions across households. Finally, when interpreting diet affordability data with respect to income, readers should be aware that Australians living in very remote areas are five times more likely to be receiving welfare payments (17% in very remote areas compared to 3.4% in major cities). Hence, given the limitations associated with diet affordability calculated relative to median income in this study, diet affordability relative to welfare income is suggested as the more policy-relevant metric. Limitations notwithstanding, results confirm that household income plays an important role in diet affordability and could, therefore, influence health and wellbeing outcomes [17]. Findings suggest that diet affordability is nuanced and sensitive to income change across Queensland regions.

Food stress and affordability thresholds (diet cost >25% and >30% of household income, respectively) were used in this study as well-accepted benchmarks. However, the authors suggest caution when applying these benchmarks as categorical indicators. This is because different thresholds could meaningfully alter the categorization of ‘food stress’ and ‘food is unaffordable’. In this study, a formal sensitivity analysis has not been undertaken, which the authors acknowledge is a limitation. Results indicate that thresholds could be altered significantly and yield similar results, particularly with respect to the food is unaffordable (30%) threshold. For example, diets costing up to 90% of the household income were classified the same as other regions in which the diet cost 31% of household income. This suggests the need for further research that refines thresholds to be more sensitive to the differing degrees of food stress/affordability. In practice, household income and expenditure are complex and influenced by structural inequity. While useful for comparisons across population groups, the food stress and affordability thresholds may not retain accuracy at the individual household or community level. In practice, households with high expenses (such as increased medical costs or high transport costs due to geographical isolation) may experience food stress/affordability issues at a lower threshold. The thresholds used in this study are not sensitive enough to reflect the real-world experiences of households who may experience food stress/affordability issues at a range of diet cost to income (%) comparisons. The food stress/affordability thresholds utilized by this study are broad, useful for informing population-level comparison and policy decisions (particularly where welfare-related metrics are concerned), but do not reflect real-world variability in household income/expenditure.

Findings of this research do not account for the strategies of Aboriginal and Torres Strait Islander communities in protecting and promoting their food security and sovereignty. For example, fishing, hunting and the collection of bush foods. These traditional practices, if supported by the broader food system, are an important opportunity for Aboriginal and Torres Strait Islander communities to exercise their human right to food and access affordable, nutritious food sources. Forced disruption of traditional food systems has created inequities related to food affordability and nutrition (particularly in remote contexts) [13,62]. Internationally, colonized Indigenous populations experience similar barriers, which underscore the need for community-led approaches to food security (supported by system-level intervention) that restore autonomy over local food systems [14,63,64].

### 4.1. Strengths and Limitations

Using one of Australia’s most widely utilized tools, this research monitored diet cost and affordability in urban, regional, and remote Queensland over two years [17]. This research adds to the few studies, globally, that have captured food pricing interventions at a sub-national level [17]. The findings have the potential to inform future health and fiscal policy actions [65].

Across Australian states and territories, food pricing and diet affordability monitoring methods are used inconsistently [17]. However, the Healthy Diets ASAP protocol has been most widely applied in Australia [17,66]. This study is strengthened by using the Healthy Diets ASAP protocol, as it is evidence-based, relevant to the study location, and utilized by other Australian researchers. However, the use of different food price/affordability monitoring methods in Australia has made direct comparisons (particularly across states and territories) difficult and highlights the need for national consistency [17].

Both diet types (healthy and habitual) were designed using the best available evidence. However, the dietary patterns investigated by this study may not reflect the actual dietary practices of families. For example, the habitual diet is constructed based on data from 2011 to 2013, which is now over a decade old. Efforts to update the Healthy Diets ASAP protocol with recent dietary intake data are encouraged [15,67]. Furthermore, this study did not measure actual purchasing behaviors nor the consumption of food/drink products upon which price data were collected.

The findings of this study suggest that healthy diet costs exceed the threshold for food stress among Aboriginal and Torres Strait Islander households, which could contribute to the risk of food insecurity. Other studies indicate that people experiencing food insecurity often cope by amending their dietary practices to skip meals, reduce portion sizes, or avoid perishable items [68]. While the diets investigated in this study are a useful benchmark for comparing over time and across regions, they may not accurately reflect the dietary patterns of Aboriginal and Torres Strait Islander families experiencing food stress. Further research that aligns price monitoring with real-life dietary practices is strongly encouraged, as is research that explores lower-cost (but still nutritionally balanced) diet options (such as pricing generic-branded items as per the Healthy Diet ASAP protocol modified for low socioeconomic groups) [3].

This study sampled 35 communities across a wide geographical area and included different store types (for example, the sample includes a mix of independently owned and store group/chain stores). While this can be considered a strength, communities included in this research were sampled through non-probabilistic methods. In remote regions, sampling was driven by existing staff travel arrangements and local partnerships that could be utilized for data collection. As a result, the sample distribution is not equal across regions and is very low in some regions (for example, in LG *n* = 2 over two years). With the exception of the LG region (in which the sample size is small), local partnerships and a network of data collectors in very remote communities supported data collection across a mostly representative sample of stores within regions, irrespective of remoteness and/or store type. In the LG region, data were collected in a small number of stores that may not have been representative of the overall store make-up/food prices within that region. Hence, diet cost and affordability data for this region (LG) should be interpreted with caution. Future research should aim to collect data from a representative sample across all regions, including a mixture of store types.

As previously established, the sample distribution (see Supplementary Table S2) highlights that food environments in remote communities are smaller and less diverse (compared to Brisbane and Cairns). Therefore, in some remote regions, prices for food/drink items (for example, alcohol) were missing/unavailable. In these cases, as per the Healthy Diets ASAP protocol, the price of the missing/unavailable items was substituted with that of the nearest regional center. This could be considered a limitation, as it does not truly reflect the local food environment, product availability, and overall diet cost in these regions. While this substitution method allows for fair comparison between regions and across years, it fails to incorporate further costs incurred by community members who may travel to obtain those items from regional centers (such as transport and fuel costs). Hence, in remote communities (where basic grocery items can be less available), regional averages may be less generalizable at the local level.

The study focuses on a six-person Aboriginal and Torres Strait Islander household. While this is representative of some households in remote FNQ regions under investigation, it may not reflect the typical household structure across Queensland or other diverse household structures. This study has assumed a uniform household structure within and across regions for the purpose of comparison. This is a limitation because in practice, household structures within and across regions are diverse. The findings are relevant to a specific reference household, limiting their broader generalizability. As such, diet cost and affordability data should be interpreted with caution, particularly in Brisbane and Cairns. Further research focused on other household structure types that may be more representative of the general population and reflect diverse household structures is recommended.

### 4.2. Implications for Policy and Practice

This study offers several important implications for diet affordability and food security policy in Queensland, Australia. Results indicate that targeted food price interventions (like the RCFAS) could be associated with reduced diet costs. However, inequities in diet affordability, and food security more broadly, persist. While the cost of healthy diets has reduced in remote communities, this represents just one determinant of food security and food choice. Food security and diet affordability are influenced by a variety of complex and interconnected factors related to structural inequities [51,64]. Diet affordability is not an equivalent measure of food security [69].

Food price interventions such as subsidies are important, but do not address the many systemic drivers of food insecurity. Further, subsidies rely on continuous government funding and, therefore, could be impractical in the long term. Addressing the underlying drivers of inequity is required for sustainable change [51,64]. For example, policy solutions that address transport and infrastructure challenges, supply chain coordination and resilience, housing, income and economic development [51,64]. These types of policy solutions, if appropriately integrated, may help overcome the systemic inequities that hold food insecurity in place in remote communities. For these reasons, multi-strategic approaches to improve food security in remote communities are recommended. This aligns with the National Strategy for Food Security in Remote First Nations Communities 2025–2035 and the Gather + Grow Strategy for Remote Food Security which advocate for coordinated, multi-pronged action [29,70].

Continued monitoring of diet affordability using standardized tools is essential. This study illustrates the impacts of food pricing interventions and highlights inequities across Queensland in their absence. Yet, Queensland and Australia lack routine surveillance systems [14]. Without such systems, policymakers and practitioners have little oversight of food pricing interventions and other food system influences. To promote evidence-based policy decision-making, grounded in equity, and a deep understanding of the food system, annual national monitoring using a consistent and up-to-date diet cost and affordability protocol is recommended. This would allow researchers to capture the influence of local and global factors on diet affordability (for example, national food pricing interventions, climate change, and supply chain instability, among others) [22,71].

Finally, health promotion efforts must reflect real-world food environments. Despite the healthy diet being more affordable (and this being a powerful public health message), there are many factors that mean this type of diet is not accessible to all. This suggests that health promotion through food price interventions is insufficient to address all the determinants of diet. This study highlights the importance of locally led and culturally safe public health strategies that respond to local barriers and are multi-strategic in nature [68]. Our findings support the commitments of the Australian Government to Closing the Gap and reinforce the role of food security as a cornerstone of health equity [71].

## 5. Conclusions

This study identified the cost and affordability of healthy and habitual diets across Queensland, focusing on a six-person Aboriginal and Torres Strait Islander household. While the healthy diet was less expensive than the habitual diet (which includes alcohol and takeaway food items), its cost still exceeded the threshold for food stress among Aboriginal and Torres Strait Islander households across Queensland. Results indicate that healthy diets have become significantly (24%) cheaper in remote Far North Queensland regions (in 2024 compared to 2023). While this study is cross-sectional, and therefore unable to identify causal factors, potential influences associated with the observed change could include the implementation of a Remote Communities Freight Assistance Scheme. In the absence of support, diets are significantly more expensive in remote communities and diet-related inequity is intensified. These findings highlight the value of ongoing monitoring using tools like the Healthy Diets ASAP protocol to track diet affordability and the impacts of food pricing and food security policy. However, diet cost and affordability reflect one dimension of food security, and broader determinants of healthy diet consumption and availability should be explored at the individual, community and food system levels.

## Figures and Tables

**Table 1:** Number of communities surveyed by region (2023 and 2024).

Region	Communities (*n*)		
	2023	2024	Number of Unique Communities Surveyed
Brisbane	2	2	2
Cairns	2	2	2
Cape York	7	9	9
Lower Gulf	2	1	2
Inner Torres Strait Islands and Northern Peninsula Area	4	6	6
Outer Torres Strait Islands	13	14	14
Total	30	34	35

**Table 2:** Estimated differences in diet cost by year, location, and diet type (*n* = 128).

	Mean Difference ($)	Standard Error (SE) ($)	*p*
Difference in diet cost across years (difference = 2024–2023)			
Non-remote			
Healthy Diet	38.7	6.8	0.000
Habitual Diet	26.7	27.4	0.338
Remote			
Healthy Diet	−321.8	19.6	0.000
Habitual Diet	−209.7	27.2	0.000
Difference in cost comparing across remote and non-remote areas (difference = remote − non-remote)			
2023			
Healthy Diet	323.8	18.2	0.000
Habitual Diet	505.3	27.9	0.000
2024			
Healthy Diet	−36.7	17.7	0.045
Habitual Diet	268.9	26.1	0.000
Difference in cost comparing across diet types (difference = healthy diet − habitual diet)			
Non-remote			
2023	−299.2	6.8	0.000
2024	−287.2	29.2	0.000
Remote			
2023	−480.7	17.8	0.000
2024	−592.8	14.4	0.000

Post hoc comparisons after linear regression with cluster robust standard errors.

**Table 3:** Mean diet affordability (diet cost relative to household income expressed as percentage (%)) in Queensland for a six-person Aboriginal and Torres Strait Islander household (*n* = 35).

Region		2023		2024	
	Income Type	Healthy Diet	Habitual Diet	Healthy Diet	Habitual Diet
Cape York	Welfare * Median **	38% 50%	48% 63%	29% 38%	44% 58%
Lower Gulf	Welfare Median	39% 52%	53% 70%	27% 36%	47% 62%
Outer Torres Strait Islands	Welfare Median	40% 65%	55% 90%	29% 48%	45% 75%
Inner Torres Strait Islands & Northern Peninsula Area	Welfare Median	40% 44%	54% 61%	29% 33%	47% 53%
Capital City	Welfare Median	31% 26%	40% 34%	31% 27%	38% 32%
Regional City	Welfare Median	30% 30%	39% 39%	30% 30%	39% 39%

>25% exceeds the threshold for food stress (denoted by light grey shading); >30% exceeds the threshold for diet affordability (denoted by dark grey shading). * Welfare income represents the household income based solely on applicable welfare payments, estimated using Department of Human Services data [38]. ** Median household incomes were derived from the 2021 Australian Bureau of Statistics Census data for each local government area (using the Census All Persons QuickStats function) and are not specific to Aboriginal and Torres Strait Islander households [39].

## Data Availability

De-identified data is available upon request.

## Supplementary Materials

The following supporting information can be downloaded at https://www.mdpi.com/article/10.3390/ijerph23040535/s1: Table S1: Fortnightly regional welfare and estimated median incomes ($ AUD per fortnight); Table S2: Distribution of food/drink outlets.

## Abbreviations

The following abbreviations are used in this manuscript:

Healthy Diets ASAP	Healthy Diets Australian Standardised Affordability and Pricing
FNQ	Far North Queensland
CY	Cape York
LG	Lower Gulf
OTSI	Outer Torres Strait Islands
TSI & NPA	Inner Torres Strait Islands and Northern Peninsula Area
