# Supplementary material for: Diet Cost and Affordability in Queensland: A Two-Year Cross-Sectional Study
Source: Int J Environ Res Public Health. 2026 Apr 20;23(4):535. doi: 10.3390/ijerph23040535 (PMC13115860; doi:10.3390/ijerph23040535)
Supplement: Supplementary file 1 [file ijerph-23-00535-s001.zip › ijerph-4240080-supplementary.pdf]

Table S1. Fortnightly Regional Welfare and Estimated Median Incomes ($ AUD per fortnight)

**Region**	**2023**		**2024**	
	**Welfare[1]**	**Median[2]**	**Welfare[1]**	**Median[2]**
Cape York	$3489.08	$2653.00	$3,630.08	$2731.00
Lower Gulf	$3489.08	$2641.00	$3,630.08	$2719.00
Outer Torres Strait Islands	$3489.08	$2122.00	$3,630.08	$2184.00
Inner Torres Strait Islands & Northern Peninsula Area	$3489.08	$3125.00	$3,630.08	$3217.00
Capital City	$3420.38	$4005.00	$3,561.38	$4212.00
Regional City	$3420.38	$3431.00	$3,561.38	$3532.00

[1]Welfare incomes are taken from Department of Human Services data about welfare allowances for an Aboriginal and Torres Strait Islander household and include the remote area allowance where relevant. Welfare income was calculated based on the following assumptions for a six-person Aboriginal and Torres Strait Islander household; the adult male and female are partnered parents of the three dependent children, adult male is unemployed and looking for work, adult female is a stay-at-home mum, older female receives age pension, the older children attend school and are fully immunized, the youngest child attends kindergarten 2 days/week and is fully immunized, the family does not have savings or investments, and the family is living in public housing.

[2]Median incomes estimation are based on data from the 2021 Census data (with a Wage Pricing Index adjustments) and are not specific to Aboriginal and Torres Strait Islander Households.

Table S2. Distribution of Food/Drink Outlets

Region		Number of Food/Drink Outlets Surveyed	
		2023	2024
Brisbane	Supermarket	6	6
	Bakery	2	2
	Fish and Chips Shop	2	2
	Burger restaurant	2	2
	Liquor store	2	2
	Pizzeria	2	2
	**Total**	**16**	**16**
Cairns	Supermarket	5	5
	Bakery	2	3
	Fish and Chips Shop	2	2
	Burger restaurant	2	2
	Liquor store	2	2
	Pizzeria	2	1
	**Total**	**15**	**15**
CY	Supermarket	7	9
	Bakery	3	4
	Fish and Chips Shop	1	4
	Burger restaurant	3	3
	Liquor store	1	2

	Pizzeria	2	2
	**Total**	**17**	**24**
LG	Supermarket	2	1
	Bakery	1	1
	Fish and Chips Shop	1	1
	Burger restaurant	1	1
	Liquor store	1	0
	Pizzeria	1	1
	**Total**	**7**	**5**
TSI & NPA	Supermarket	4	8
	Bakery	2	3
	Fish and Chips Shop	3	0
	Burger restaurant	3	0
	Liquor store	2	2
	Pizzeria	3	0
	**Total**	**17**	**13**
OTSI	Supermarket	13	14
	Bakery	0	1
	Fish and Chips Shop	0	0
	Burger restaurant	0	0
	Liquor store	0	0
	Pizzeria	0	0
	**Total**	**13**	**15**
**TOTAL**		**85**	**88**
